# Supplementary material for: Pan-Genome Analysis Reveals Host-Specific Functional Divergences in Burkholderia gladioli
Source: Microorganisms. 2021 May 22;9(6):1123. doi: 10.3390/microorganisms9061123 (PMC8224644; doi:10.3390/microorganisms9061123)
Supplement: Supplementary file 1 [file microorganisms-09-01123-s001.zip › microorganisms-1208102-supp/Figure S1 and Table S2.pdf]

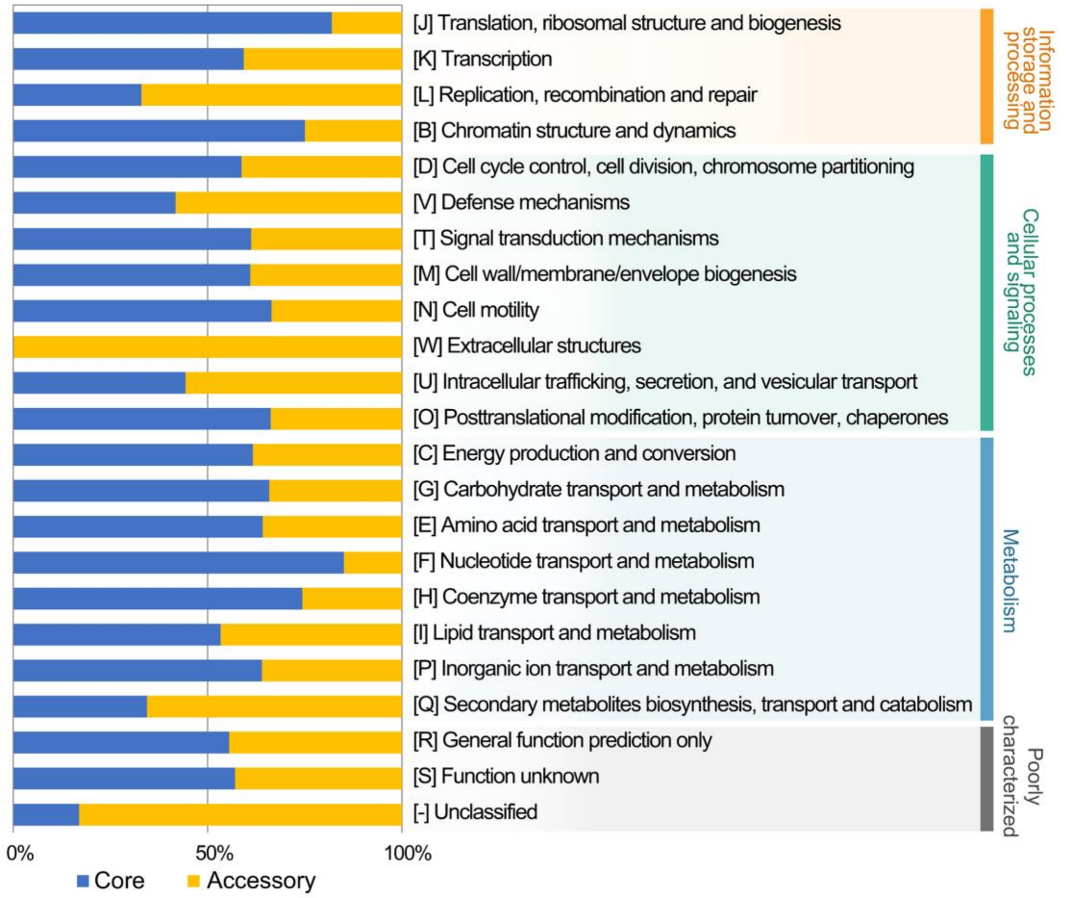

Figure S1. Clusters of orthologous group (COG) distribution of the Burkholderia gladioli core and accessory genomes.

Table S2. GO enrichment analysis of *Burkholderia gladioli* core genomes.

GO ID	Term	Class	Count	*P* value
GO:0005737	Cytoplasm	C[a]	2877	0.00.E+00
GO:0005840	Ribosome	C	597	0.00.E+00
GO:0005887	Integral component of plasma membrane	C	343	2.14.E-12
GO:0015627	Type II protein secretion system complex	C	142	8.47.E-11
GO:0005886	Plasma membrane	C	2418	3.83.E-10
GO:0015934	Large ribosomal subunit	C	98	1.13.E-07
GO:0009425	Bacterial-type flagellum basal body	C	93	2.56.E-07
GO:0042597	Periplasmic space	C	214	3.80.E-07
GO:0015935	Small ribosomal subunit	C	83	1.31.E-06
GO:0032153	Cell division site	C	74	5.71.E-06
GO:0005524	ATP binding	F[b]	3024	0.00.E+00
GO:0046872	Metal ion binding	F	1873	0.00.E+00
GO:0003735	Structural constituent of ribosome	F	734	0.00.E+00
GO:0019843	rRNA binding	F	467	0.00.E+00
GO:0005525	GTP binding	F	311	0.00.E+00
GO:0000049	tRNA binding	F	206	2.44.E-15
GO:0003924	GTPase activity	F	196	1.22.E-14
GO:0051539	4 iron, 4 sulfur cluster binding	F	388	1.62.E-12
GO:0008270	Zinc ion binding	F	524	4.21.E-10

GO:0004888	Transmembrane signaling receptor activity	F	120	3.10.E-09
GO:0006412	Translation	P[c]	818	0.00.E+00
GO:0006935	Chemotaxis	P	224	1.11.E-16
GO:0006457	Protein folding	P	206	2.44.E-15
GO:0051301	Cell division	P	206	2.44.E-15
GO:0071973	Bacterial-type flagellum-dependent cell motility	P	183	1.03.E-13
GO:0007165	Signal transduction	P	160	4.45.E-12
GO:0016310	Phosphorylation	P	459	4.45.E-12
GO:0007049	Cell cycle	P	152	1.65.E-11
GO:0071555	Cell wall organization	P	226	4.21.E-11
GO:0015628	Protein secretion by the type II secretion system	P	142	8.47.E-11

[a] Cellular component; [b] Molecular function; [c] Biological process
